# Endoscope-assisted resection of a Samii type B jugular foramen schwannoma

**DOI:** 10.1007/s00701-022-05481-7

**Published:** 2023-01-12

**Authors:** Xin Zhang, Hao Xu, Wei Hua, Wei Zhu

**Affiliations:** 1 Department of Neurosurgery, Huashan Hospital, Fudan University, Shanghai, 200040 China; 2 National Center for Neurological Disorders, Shanghai, 200040 China; 3 Shanghai Key Laboratory of Brain Function Restoration and Neural Regeneration, Shanghai, 200040 China; 4 Neurosurgical Institute of Fudan University, Shanghai, 200040 China; 5 Shanghai Clinical Medical Center of Neurosurgery, Shanghai, 200040 China

**Keywords:** Jugular foramen, Schwannoma, Endoscope, Lower cranial nerve, Neurosurgery

## Abstract

**Background:**

Tumors involving the jugular foramen region are challenging for surgical resection. With the development of endoscope in the past decade, surgical approaches assisted by endoscope have been widely emerged in the treatment of skull base tumors.

**Methods:**

Herein, we report a case of jugular foramen schwannoma (Samii type B). Surgical resection was applied via a suboccipital retrosigmoidal craniotomy using surgical microscope assisted by endoscope. Gross total resection was achieved. And the patient recovered without obvious neurological deficits.

**Conclusions:**

Samii type B schwannomas involving the jugular foramen is approachable by endoscope-assisted surgery.

**Supplementary Information:**

The online version contains supplementary material available at 10.1007/s00701-022-05481-7.

## Relevant surgical anatomy

Jugular foramen schwannomas (JFS) constitute 2.9–4% of intracranial schwannomas, which are the third common benign brain tumors [1]. Radical resection is the primary neurosurgical goal for this kind of complicated skull base tumor if possible, and the preservation of the lower cranial nerves (CN IX, X, XI, and XII) is challenging yet mandatory. Stereotactic radiosurgery (SRS) may provide efficient control of irresectable JFS similar to vestibular schwannomas (VS), and 14.3% of tumor progression is observed after long-term follow-up not in VS [5].

Jugular foramen region is involved with several important neurovascular structures, such as the lower cranial nerves and jugular vein. An optimal neurosurgical approach is critical to achieve total resection of the tumor. According to tumor location and extensions, JFSs are classified into three subtypes by Samii, which are type A (tumors mainly in the cerebellopontine angle with enlargement of jugular foramen), type B (tumors mainly in the foramen with intracranial extension), and type C (mainly extracranial tumors with extension in the jugular foramen and dumbbell-shaped tumors in both the intracranial, jugular foramen and extracranial compartment) [4]. Numerous approaches including the far lateral approach, juxtacondylar approach, and postauricular transtemporal approach, have been introduced based on this classification [2]. A two-piece lateral suboccipital approach could be appropriate for extensive dumbbell shaped JFS [6].

Recently, endoscopic far medial approach has been reported for the resection of the tumors in the jugular foramen region [3, 7]. Endoscope-assisted technique has shown significant advantages in skull base surgery regarding radical resection of skull base tumors. We have reported the endoscope-assisted resection of the middle cranial fossa cholesteatomas in the keyhole craniotomy [8]. Advanced endoscope-assisted microscopy surgeries can provide more alternative approaches during the resection of JFS with less invasive craniotomy and fewer neurological deficits.

## Description of the technique

### Case description

**Fig. 1:**
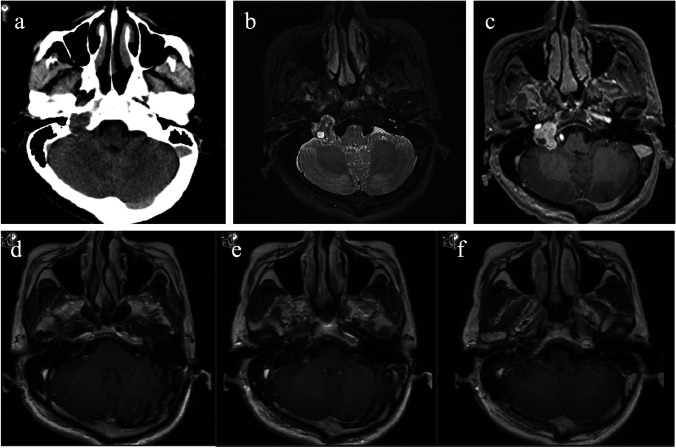
Pre-operative and post-operative radiological images. **a** Pre-operative CT shows a iso-density lesion located in the right jugular foramen region. **b and c** MRI indicates the lesion is iso-signal in T2-weighted image, and homogenous enhancement after contrast, a Samii type B schwannoma is highly suspected. **d–f** Post-operative MR confirms total resection of the tumor

A 54-year-old man presented with dizziness for 3 months before admission to the hospital. Neurological examination revealed positive pharyngeal reflex and that the tongue leaned to the middle. The patient showed not obvious sensory or motor deficits. Computed tomography (CT) showed an iso-density mass located in the right jugular foramen region, with foraminal enlargement. Magnetic resonance imaging (MRI) indicated that the lesion could be heterogeneously enhanced with cystic nodules (Fig. 1). JFS was highly suspected. Since the majority of the lesion was located within the foramen, with extension to the intracranial cistern, the lesion was classified as Samii type B schwannoma.

## Positioning and room set-up

The patient was placed in a left lateral position. Intraoperative neurophysiological monitoring (IONM) of CN VII, IX, X XI, and XII was applied to the patient. Neuro-navigation was performed routinely. Surgical microscope (P900, Zeiss) and endoscope (Karl Storz, Germany) were both prepared for the operation.

## Tumor resection using microscope

Right suboccipital retrosigmoidal craniotomy was performed as routine. The dura was cut in a radiated fashion. After the magnum foramen cistern was opened and cerebrospinal fluid (CSF) was released, the tumor was exposed as satisfied intracerebral pressure was achieved. Then the intracranial part of the tumor was resected in a piecemeal fashion. IONM of the lower cranial nerves was applied via an electrode during the resection, in order to identify and preserve the nerves from the tumor.

## Endoscopic resection

**Fig. 2:**
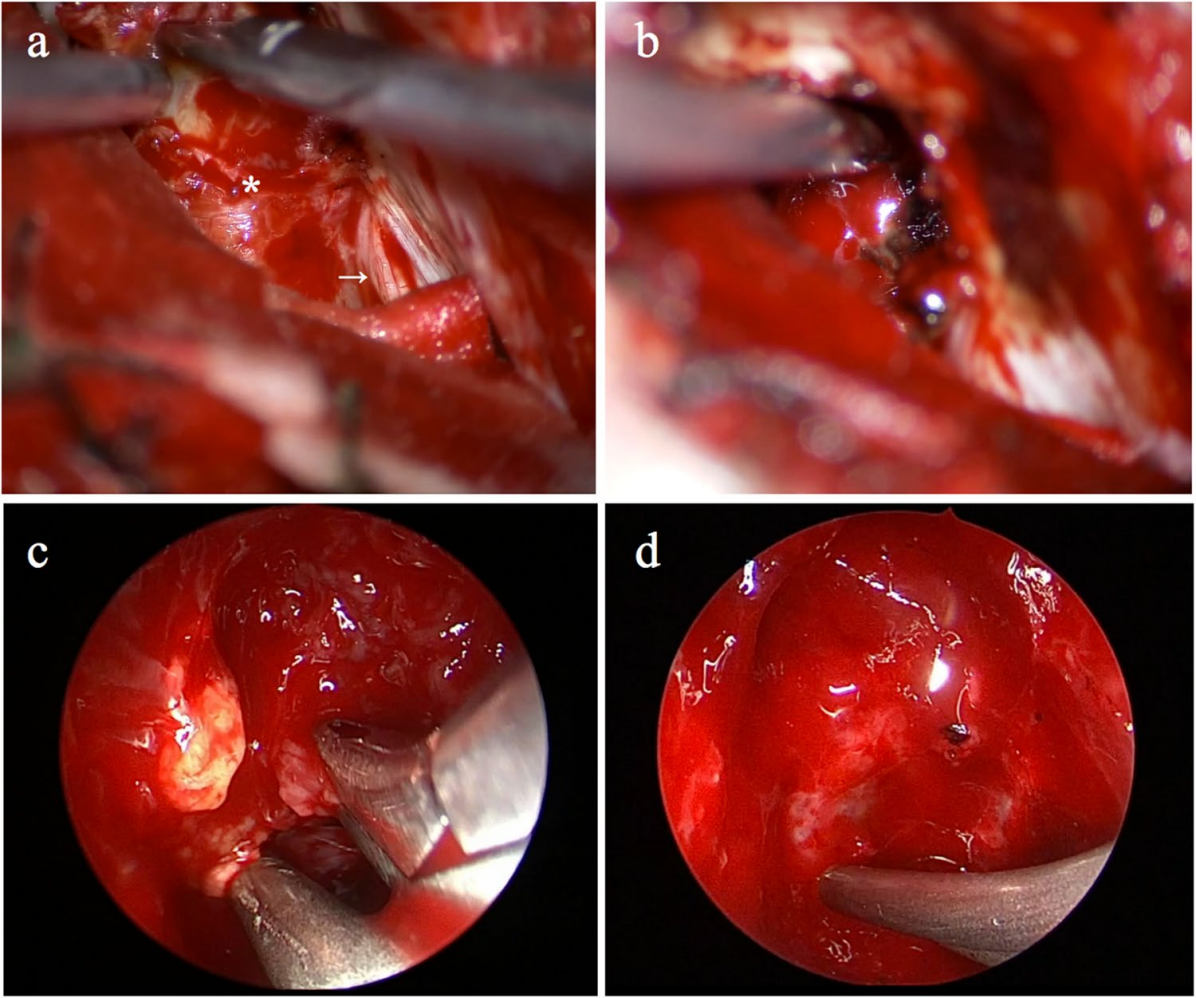
Intraoperative images. **a** Tumor (asterisk) located in the jugular foramen region is exposed, superior to the lower cranial nerves (white arrow). **b** Microscopic view after the tumor is removed under the microscope to the largest extent. **c** View of 45-degree endoscope indicates some remnant in the superior and medial aspects of the jugular foramen. **d** Endoscopic view after the tumor is totally removed

After the intracranial tumor was removed, a rigid 45-degree endoscope (Karl Storz, Tuttlingen, Germany) was applied for further resection of the residue tumor within the jugular foramen. Endoscope could be driven by the assistant or be clamped via a pneumatic autoholding system carefully diving into the foraminal region. Gelfoam and cottons were covered on the surface of the cerebellum to minimize the brain contusion from the scope. The remnant tumor in the foramen was directly observed. Angled curette and ethmoid forceps were used to detach and remove the tumor in a piecemeal fashion. The distal carrier nerve was identified, coagulated with bipolar and then cut. Eventually, the tumor in the foramen was totally resected. Surgicel and bioglue were applied in the jugular foramen to prevent hemorrhage inside (Fig. 2).

## Post-operative MRI and evaluation of the cranial nerve function

MRI was performed within 72 hours after the surgery. The extent of resection was evaluated in the post-operative MRI, which was confirmed as gross total resection of the tumor both in the jugular foramen and intracranially.

Cranial nerve functions were evaluated by the neurosurgeons after surgery. No dysphagia and hearing loss were observed in this patient. The patient was discharged uneventfully.

## Indications

This approach assisted by the endoscope is suitable for tumors located in and involved with the jugular foramen. Traditional microscopic surgery has access to remove the intracranial part of the tumor, however, is left with intraforaminal remnant in many cases. Herein, with the help of angled endoscope and instruments, we not only have a better view into the jugular foramen but also have the capacity to remove the part of the tumor located in the jugular foramen. Surgical approaches may change with the advent of new techniques.

## Limitations

In cases of tumors with much extracranial extension, this technique may be inadequate for lateral exposure and maneuver of the endoscopic instruments. Fisch approach is suitable for type C JFS. For tumors with significant adhesion to the lower cranial nerves, gross total resection may not be able to achieve to avoid neurological deficits. Subtotal resection followed by radiosurgery is a reasonable option under this circumstance. Recently, a new model of microscope with Qevo (Zeiss, Oberkochen, Germany) endoscope has been introduced, which combines microscope and endoscope for better visualization during surgical procedures. With the help of Qevo angled endoscope, tumors within the jugular foramen might be resected more easily without residue.

## How to avoid complications

The major complication of this technique is cerebellar contusion due to contact of the endoscope and the brain. As a result, an autoholder is recommended because of its stability. Fixation arm of the autoholder should be used to reduce the cerebellar contusions.

Schwannomas involving the jugular foramen region are supposed to originate from lower cranial nerves. Complications associated with lower cranial nerve deficits include dysphagia and voice hoarseness, which are critical to the outcome and life quality. Prediction of the lower cranial nerve origin before the surgery is difficult. However, we routinely perform IONM to identify the nerves and minimize the neurological deficits intraoperatively.

Direct trauma or indirect thermal injury are other considerable complications with this technique.

## Specific information for the patient

The patient should be fully informed of the risks of the surgery, such as hemorrhage, infection, lower cranial nerve deficits, and cerebellar edema.

## Supplementary Information

Below is the link to the electronic supplementary material.

Supplementary file1 (MP4 74961 KB)

## Data Availability

The clinical information and surgical video are approved by the patient.
